# Calibration and validation of a novel hybrid model of the lumbosacral spine in ArtiSynth–The passive structures

**DOI:** 10.1371/journal.pone.0250456

**Published:** 2021-04-26

**Authors:** Robin Remus, Andreas Lipphaus, Marc Neumann, Beate Bender

**Affiliations:** 1 Chair of Product Development, Department of Mechanical Engineering, Ruhr-University Bochum, Bochum, Germany; 2 Biomechanics Research Group, Chair of Product Development, Department of Mechanical Engineering, Ruhr-University Bochum, Bochum, Germany; National University of Ireland Galway, IRELAND

## Abstract

In computational biomechanics, two separate types of models have been used predominantly to enhance the understanding of the mechanisms of action of the lumbosacral spine (LSS): Finite element (FE) and musculoskeletal multibody (MB) models. To combine advantages of both models, hybrid FE-MB models are an increasingly used alternative. The aim of this paper is to develop, calibrate, and validate a novel passive hybrid FE-MB open-access simulation model of a ligamentous LSS using ArtiSynth. Based on anatomical data from the Male Visible Human Project, the LSS model is constructed from the L1-S1 rigid vertebrae interconnected with hyperelastic fiber-reinforced FE intervertebral discs, ligaments, and facet joints. A mesh convergence study, sensitivity analyses, and systematic calibration were conducted with the hybrid functional spinal unit (FSU) L4/5. The predicted mechanical responses of the FSU L4/5, the lumbar spine (L1-L5), and the LSS were validated against literature data from in vivo and in vitro measurements and in silico models. Spinal mechanical responses considered when loaded with pure moments and combined loading modes were total and intervertebral range of motions, instantaneous axes and centers of rotation, facet joint contact forces, intradiscal pressures, disc bulges, and stiffnesses. Undesirable correlations with the FE mesh were minimized, the number of crisscrossed collagen fiber rings was reduced to five, and the individual influences of specific anatomical structures were adjusted to in vitro range of motions. Including intervertebral motion couplings for axial rotation and nonlinear stiffening under increasing axial compression, the predicted kinematic and structural mechanics responses were consistent with the comparative data. The results demonstrate that the hybrid simulation model is robust and efficient in reproducing valid mechanical responses to provide a starting point for upcoming optimizations and extensions, such as with active skeletal muscles.

## Introduction

Low back pain affects more than 80% of all adults over the course of a lifetime [1] and is therefore by far the most common diagnosis for musculoskeletal disorders in Germany [2]. Only the physiological interaction of muscles with the passive and primarily load-bearing structures of the spinal column ensures their stability while enabling mobility under high varying loads [3, 4]. Due to the complexity of the spine and neural mechanism of chronic pain, causes of back pain are often difficult to identify and are not yet fully understood [5, 6]. Etiologies can be biomechanical in nature like minor instabilities of the musculoskeletal system [7] or small structural pathologies [8]. A variety of surgical and conservative measures exist to treat the condition [9, 10], whose efficacy could be further improved by expanding the biomechanical understanding [11, 12]. However, even latest experimental methods are reaching their limits in studying and treating the causes of pain [13]. In vitro, physiological load conditions can be simulated to a limited extent only [14, 15]. In vivo, the possibilities of investigation, for example regarding muscle forces, the exact kinematics or pain and stress states in soft tissues are limited, costly or not ethically justifiable [13, 16, 17]. To extend functional knowledge non-invasively and improve the effectiveness of treatments, two computational biomechanics methods, musculoskeletal multibody (MB) [18–21] and finite element (FE) methods [22–29], have been intensively used over the last decades. The differences between the two numerical simulation methods are that the MB method is generally used to analyze mechanisms consisting of rigid bodies subject to joints and constraints. FE analyses, on the other hand, allow a detailed investigation of the structural behavior when, for instance, the deformations or stresses are of interest. Using mainly these two methods a wide range of lumbar spine models with variant scopes, details, and objectives exist in the scientific literature [30].

Common applications for multibody simulations include the computation and study of interindividual muscle activity patterns, joint reaction forces, or vertebral movements in varying postures and activities, to gain insight into the potential biomechanical differences of persons without and with low back pain [31, 32]. Musculoskeletal MB models consist of rigidly assumed bones interconnected by joints, constraints, as well as tensile muscles and general spring-damper elements (ligaments) [30, 33]. When a muscle redundancy problem needs to be solved to obtain the muscle activations used to describe a given kinematics, an inverse simulation approach can be distinguished from a forward or direct simulation approach [34]. Joint descriptions for intact functional spinal units [31, 35–38] as well as for the intervertebral disc only [39, 40] may, however, limit the capabilities of the analysis for an interaction between muscle forces and joint kinematics [41, 42]. Basic joints are represented by spherical or ball joints with three degrees of freedom and without torque transmission [19, 32, 37, 43, 44]. Physiological vertebral movements usually occur not only in the force direction, but also as coupled movements in other directions [45]. Therefore, shear deformable FE beam elements [38] and various mostly linearized custom joint approaches with six degrees of freedom and 6x6 stiffness matrix formulations [46–49] are used for a more detailed replication of the intervertebral passive joint structures. However, due to lacking experimental data, off-diagonal terms of the joint stiffness matrices may be incomplete [48], whereby physiological motion couplings often cannot be fully represented [37, 47, 50]. Current rotational motions of vertebrae in space are described by instantaneous axes of rotation (IAR) [51]. For planar movements, IAR can be simplified by instantaneous centers of rotation (ICR), defined by the points where the IAR intersect the movement plane [52, 53]. Zander et al. [54] have shown that the positions of defined joint rotation centers have a considerable effect on muscle forces and lumbar loads. Joint descriptions with six degrees of freedom are not limited in terms of locally traveling ICR compared to spherical joints, but their kinematics are still sensitive to the initial positioning [52]. Overall, usually computational efficient MB models are primarily used in context of spine dynamics, but are limited in determining the load distribution between discs, facet joints, and ligaments as well as directly calculating intradiscal pressures [55].

For discretization and simulation of mechanical systems, the finite element method is an alternative to MB simulations. Amongst others, implicit FE studies using a direct approach aim to evaluate the intradiscal pressure [56], the load sharing [57], the bulging of the intervertebral disc, as well as the coupling of movement [58] or the contact forces in the facet joints [59] under different loads in physiological or pathological condition. Those studies show that the results agree well with in vitro measurements. In common, however, implicit FE models usually neglect probable in vivo influences of muscle forces and body weight [55, 60–62] and simplify them by a compressive follower load (FL) combined with a pure moment [57, 63–67]. The FL is a force that follows the lumbar lordosis and approximately passes in the sagittal plane through the vertebral body centers, resulting in minimal rotations of the vertebrae and preventing the spine from buckling under high compression [14]. Due to this idealization, shear force components and sagittal compression variations caused by partially high muscle forces cannot be examined [68].The consideration of numerous (passive) deformable components as well as contact problems contribute to an increased computational effort in implicit FE simulations and unfortunately often limit the computational scope to static, quasi-static or only short simulation sequences [39, 60, 62, 69].

Many clinical problems, however, span both methods. To overcome the described hurdles and to improve the quality of the results, two separate spine models have therefore been coupled to use the results of the other model as complementary input data. For instance, Weisse et al. [50] intended to use a detailed functional spinal unit FE model for stiffness matrix parameter determination to define the mechanics of an intervertebral multi-body joint more precisely. Shirazi-Adl et al. [38] coupled a simplified beam-rigid body model containing (active) lumbar muscles, which is used for efficient muscle strength calculation and optimization, with a ligamentary spine model. State of the art is the non-concurrent [55, 70, 71] or stepwise iterative [68, 72] solution of two lumbar spinal models similar in their anatomy and mechanical response. Initially, combined loading modes are calculated by means of a musculoskeletal MB model and applied to the passive elements of an implicit FE model for a subsequent detailed structural mechanical analysis. Due to that, internal strains and stresses can be simulated under complex combined loading modes that mimic probable in vivo loads. If the data transfer is not manual, the models set up in different programs must be linked via an interface that is often ambitious. Khoddam-Khorasani et al. [68] and Liu et al. [70] describe the challenges of adjusting the biomechanical responses of both models under similar loads. Overall, the interaction between the passive spinal components with active skeletal muscles represents a complex, interdependent relationship, which cannot yet be fully investigated even with a staggered coupling of simulations using MB and FE models. Iterative solutions overcome this inaccuracy, but for each calculation step both models must be solved and aligned until their solutions converge [68].

Hybrid FE-MB model simulations are a hitherto less established way to overcome the drawbacks of musculoskeletal MB and implicit FE models without couplings. Passive hybrid models are characterized by the combination of rigid and elastic FE bodies and by the fact that even complex biomechanical systems with soft tissues requiring large deformations can be dynamically calculated with great computational efficiency [38, 60, 73, 74]. Current passive hybrid models of the lumbar spine have been built in explicit FE environments (e.g. ABAQUS/Explicit) [69, 75–77]. By using explicit integration of the given differential equations, accurate results can be obtained in a stable and computationally efficient manner if the time steps are sufficiently small and the system behaves comparatively soft [34, 73]. Shirazi-Adl [78] presented an early passive hybrid spinal model for basic static biomechanical investigations already in 1994 in a dedicated nonlinear FE environment [79]. The current motivation for hybrid spine modeling is primarily the reduction of computational time, with the resulting advantages for a simplified model structure and increased usability in clinical routine [69, 75–77, 80, 81]. If a hybrid model also includes force actuators (muscles), it can be called an active hybrid model [60]. In other biomechanical research fields, the advantages of active hybrid models are often used, for example, in knee [73, 82] or jaw-tongue-hyoid language simulations [74]. To the best of our knowledge, only Knapik et al. [62] have introduced an active hybrid model of the lumbosacral spine. This personalized basic model is focused on the effects of a total disc replacement at L5/S1 level and reveals ranges of motion altered by the procedure. Limitations mentioned by the authors are primarily based on the multi-body dynamic simulation environment (MSC ADAMS) used: For example, for the creation of the flexible discs, an upstream normal mode analysis with a pre-load in neutral erect stand had to be conducted in Nastran (MSC Software). Furthermore, muscles are solely represented by a series of EMG driven force vectors and the model itself is not freely available to be shared with the community. Overall, however, active hybrid models build on an underlying passive load-bearing base structure that has particular relevance for biomechanical validity.

The aim of this paper is to address the discussed hurdles by developing, calibrating, and validating a passive hybrid simulation model of the ligamentous lumbosacral spine (LSS) from scratch, in order to join the advantages of established MB and implicit FE models and encourage open science in the field. A computational evaluation of the mechanical responses of the spine under realistic loading conditions is important, since experiments with patients are limited and poorly understood variations may be signals for pathologies potentially leading to low back pain. Therefore, the relevant mechanical responses to be investigated are total and intervertebral range of motions, instantaneous axes and centers of rotation, facet joint contact forces, intradiscal pressures, disc bulges, and stiffnesses. However, a comprehensive classification of the objective can only be made against the background that the passive LSS is intended to be the basis for a comprehensive active simulation model of the middle and lower trunk and must therefore be subordinately characterized by its robustness, simplicity, and efficiency. However, the upcoming upgrade by adding skeletal muscles as tensile actuators is not part of this paper.

## Materials and methods

The Java-based open source framework ArtiSynth (www.artisynth.org), a physics simulator that supports the combined simulation of MB and FE models [34], is used to realize the hybrid LSS simulation model. Since all degrees of freedom are primarily described by deformable bodies, the system behavior is stiff and a robust first order implicit integrator (Constrained Backward Euler) is used. The control of all simulations as well as the data recording and downstream evaluation of relevant results are carried out with Matlab (R2019b, MathWorks Inc., US) via the integrated Matlab interface [34] in ArtiSynth. For benchmarking purposes, we run our simulations and optimizations on a Dell Latitude E5550 with i7-5600U, NVIDIA GeForce 840M (2 GB VRAM), 16 GB DDR3 RAM, 512 GB Micron 1100 SATA SSD, and Windows 10 Enterprise 64-Bit.

**Fig 1:**
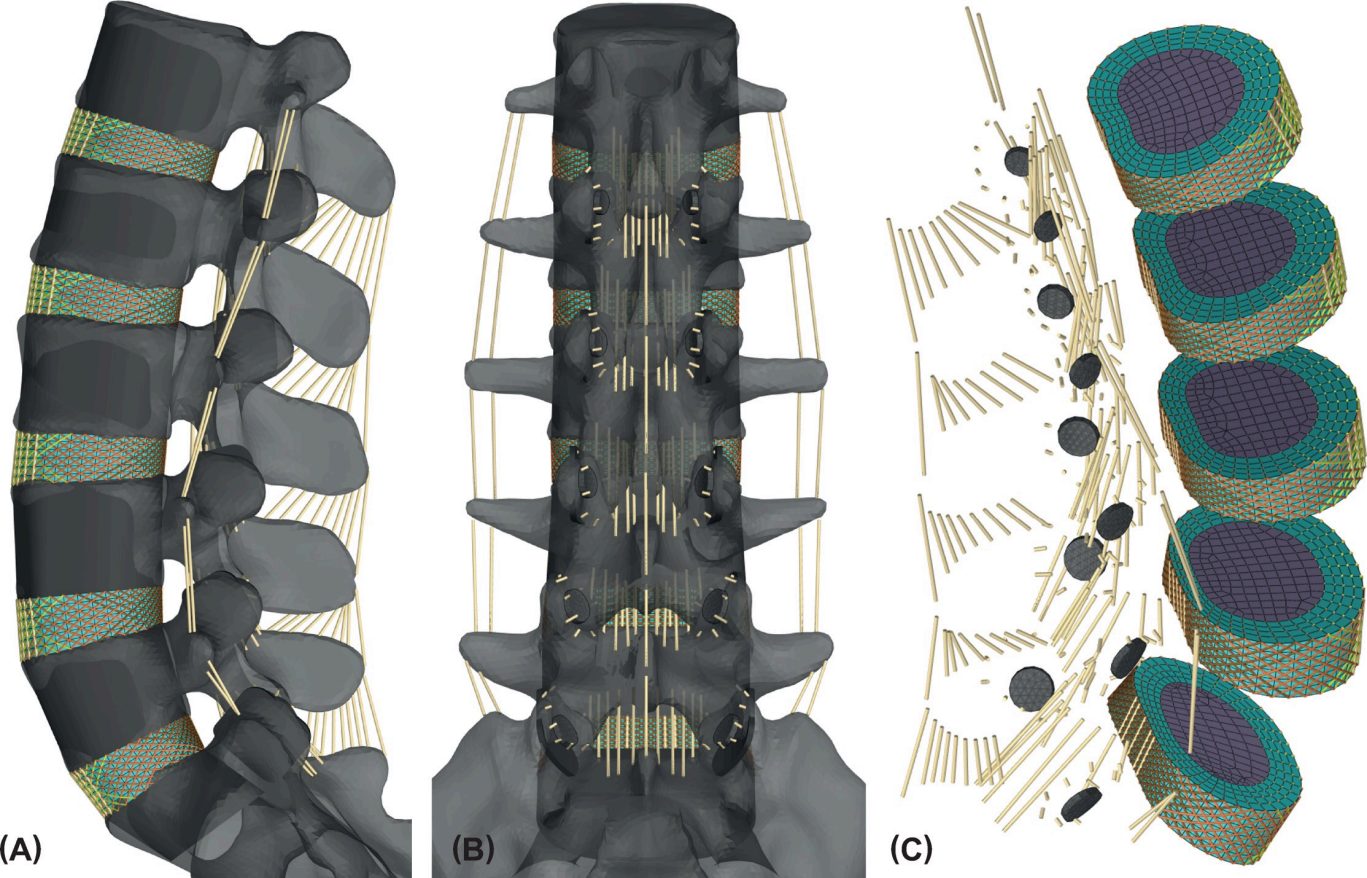
Passive hybrid lumbosacral spine model built in ArtiSynth. (A) Ligamentous LSS (L1 to sacrum) in left lateral view and (B) in dorsal view. Rigid bodies of the vertebrae are shown in light grey and auxiliary vertebral bodies together with the superior articular processes (Fig 2A) are shown in dark grey. (C) All rigid bodies are set invisible. Only the meshed inferior articular facets, the collagen fiber reinforced discs, and the ligaments are visualized. Collagen fibers assigned to different locations are distinguished by color: brown (posterior), orange (lateral), and yellow (anterior).

In the following, we cover our approach to building the passive hybrid ligamentous lumbosacral spine model (Fig 1), followed by systematic testing of the models’ mechanical responses. For better clarity, we separate the model testing into the three main sections: verification, calibration, and validation. First, we conduct a mesh convergence study and a pre-calibration in the verification section, to evaluate the L4/5 disc discretized by finite elements in the hybrid context. Using simplified L4/5 function spinal units (FSU), the pre-calibration also serves to tune and improve the initial mechanical responses, stability, and efficiency of the model, e.g. by comparing different constitutive equations. During calibration, we stepwise extend the FSU L4/5 by cumulatively adding anatomical components such as ligaments and facet joints and adjust their respective influences on range of motion according to in vitro measurements. Lastly, we validate the kinematic and structural mechanical responses of the single FSU L4/5, the lumbar spine, and the LSS with respect to various pure and combined load cases by choosing model parameters that provide the best match to the reference data. The final passive hybrid model of the LSS can be downloaded from https://doi.org/10.5281/zenodo.4453702 or https://github.com/RemusR9/artisynth_lumbosacralSpineModel.

### Passive hybrid modeling

Anatomical basis and relations are based on CT data of a 38 years old man with a height of 180.34 cm and 90.3 kg from the Visible Human Project (VHP) [83]. Only lumbar vertebrae and the sacrum are segmented and smoothed with MIMICS (v.21.0, Materialise, Belgium) from this data set. All subsequent geometric modifications and supplements (e.g. discs) are performed with ANSYS SpaceClaim (v19.2, ANSYS Inc., US), a computer-aided design software that uses solid modeling technique. Further smoothing and simplifying of the bone geometries are achieved with the facet tools implemented in ANSYS SpaceClaim. According to common practice [84], the LSS is globally oriented so that X points ventral, Y left lateral and Z cranial. The origin of the global coordinate system lies within the center of the S1 superior vertebral endplate, and the L3/4 intervertebral disc is aligned horizontally to the X-Y plane.

Since we do not focus on individual anatomical features for the time being and obtain data from datasets in which mostly no distinction is made between left and right halves of the body [18, 39, 85], we assume our model to be symmetric. For this purpose, we manually symmetrized the segmented VHP bones to the sagittal plane (corresponding to the X-Z plane). Due to the ventrodorsal weight force [86] and a settling and flattening of the tissue of the VHP body frozen in supine position [83], we assume that the lumbar lordosis is flattened in the data set. To mimic an asymptomatic lordosis in a normal upright position, we first adjusted the poses of the five lumbar vertebrae and the sacrum roughly according to literature data [87, 88]. Intersegmental relations such as the ventral height of a disc (function of the mean cranial vertebral body depth and the angle between the adjacent vertebral body mid-planes) [89] were then verified and adjusted in case of excessive deviations. Final COBB angles are 52° in between L1-S1 and 43° in between L1-L5. According to Roussouly et al. [88] the adjusted LSS is well balanced and can be classified as type 3: The apex is located at the top of L4 vertebral body, the inflection point is at the upper end of L1, the sacral slope is 36° and the lordosis tilt is 2°.

#### Rigid bodies

**Fig 2:**
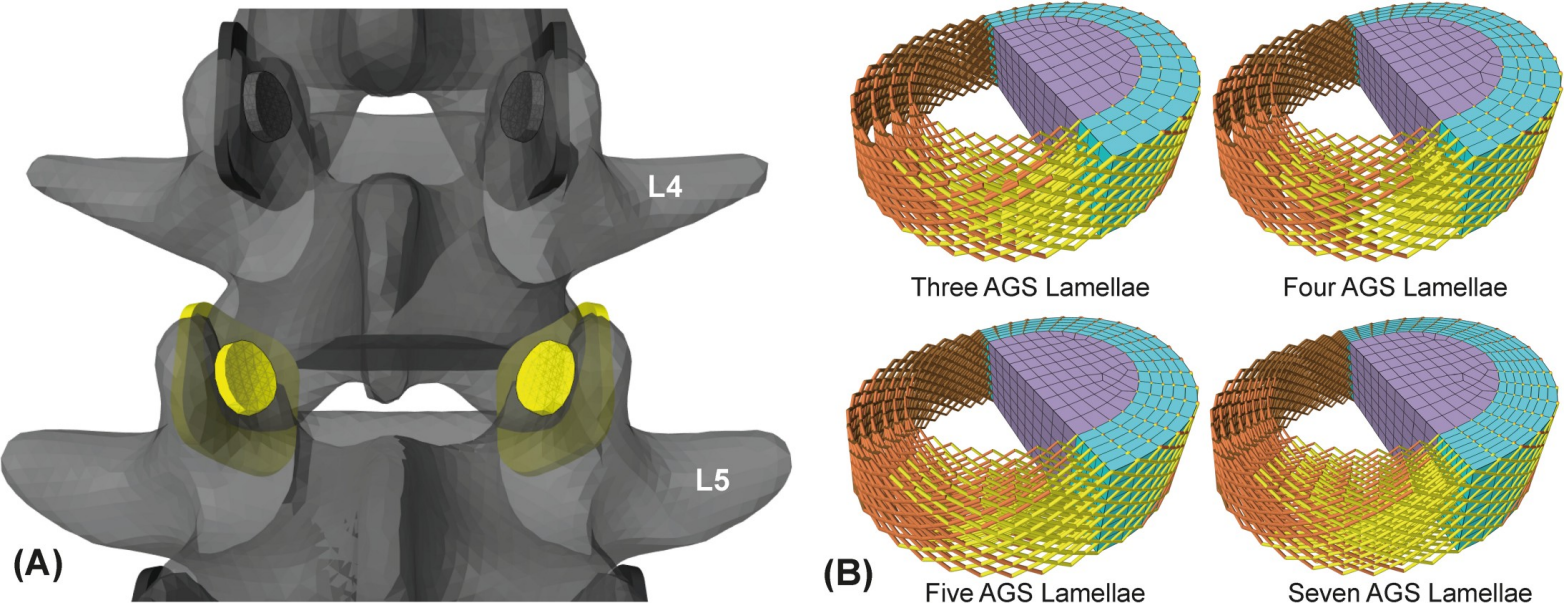
(A) Yellow highlighted L4/5 facet joints in dorsal view and (B) L4/5 disc variations. (A) Detailed view of the vertebral levels L3 to L5. Only rigid vertebrae and facet joints, which consist of the flexible inferior articular facets and rigid superior articular processes, are shown. (B) Varying disc discretizations for pre-calibration purposes. The L4/5 disc with three, four, five, and seven AGS lamellae corresponding to four, five, six, and eight CF rings is shown. FE meshes for AGS and NP are shown only for the left sides of the discs.

Vertebral bones and end plates are not differentiated and are modeled as rigid bodies. From the VHP vertebrae rigid auxiliary vertebral bodies (Fig 1A) have been derived and adapted according to anatomical data [90]. The auxiliary vertebral bodies serve for idealized planar linking surfaces to the five intervertebral discs, are massless, and are rigidly connected to the VHP vertebrae. To describe user-specific soft contacts in the facet joints, only the superior articular processes are modeled as additional rigid cylindrical auxiliary sections (Fig 2A) and are also solidly connected to the respective VHP vertebrae. The general facet joint morphology is based on the description by Kapandji and Rehart [91], so that the facet joint surfaces are arranged on cylinders with their centers located dorsally in the area of the spinous process. Precise positioning and orienting of the superior articular processes, which are hollow cylinder sections extruded outwards from the cylinders, were carried out in comparison with our specific VHP anatomy and average anatomical values using map angles determined in vitro [90, 92].

#### Finite element components

Intervertebral discs connect the vertebrae. They are hyperelastic avascular structures with six degrees of freedom [93] and can be divided into two main components: nucleus pulposus (NP) and anulus fibrosus (AF) [30]. The AF surrounds the almost incompressible NP in concentric lamellae. As a composite structure, the AF consists of an anulus ground substance (AGS) with crisscrossed collagen fibers (CF) [94]. Our simplified disc geometry is deduced directly from the surfaces of the adjacent auxiliary vertebral bodies, so that the outer surfaces represent connections of the vertebral body edges. The edges of the NP are not rounded like in vivo [95]. The NP account for a volume of 43–45% of each disc [94, 96] and their centers are shifted posteriorly by about one third of the posterior AF thickness, respectively [25]. Via the hydrostatic stress, we calculate the intradiscal pressure of a disc downstream in Matlab using the negative mean of the normal stresses of all FE nodes of a NP. All FE bodies in this study are meshed with ANSYS Workbench (v19.2, ANSYS Inc., US) and are exported and automatically prepared for the import into ArtiSynth using Matlab.

Mostly the AF is modeled by 6–16 concentric lamellae [97] with integrated layers of crisscrossed CF. By means of converging disc bulges Goel et al. [58] demonstrated that at least six radial CF layers may be sufficient. However, for the AF we do not embed the CF as a true composite structure in the AGS matrix [23], but link AGS nodes of the lamellae with multi-point springs. Discs AGS lamellae are arranged in assemblies and meshed with eight-node hexahedral elements (Hex8). Subsequently, the assemblies are combined to one FE body in ArtiSynth using *FemFactory*.*addFem()*. The L4/5 disc has four AGS lamellae and with NP it consists of 1716 Hex8 elements (Figs 1C and 2B). Each lamella is one element thick and six elements high.

A two-parameter Mooney-Rivlin model with strain energy function *W* and the constants *c*_01_, *c*_10_, and bulk modulus *k* (Table 1) approximates the homogeneously assumed, almost incompressible behavior of the AGS [26, 98]:

$$W=c_{01}(I_{1}-3)+c_{10}(I_{2}-3)+k{\left(J-1\right)}^{2}$$ (1)

To represent the nonlinear stiffening behavior of the intervertebral disc under high compression, the 3^rd^ order polynomial Yeoh material model [99] is used for the final NP model:

$$W=\sum_{i=1}^{3}c_{i0}{\left(I_{1}-3\right)}^{i}+k\sum_{i=1}^{3}{\left(J-1\right)}^{2i}$$ (2)

For initial facet gap widths, there is no generally valid size [59]. Common values vary between 0.5 and 1.5 mm [59, 70, 100]. We use a distance of 0.5 mm between the concentric facet joint surfaces and define the contact as frictionless, allowing only compression forces to be transmitted [100–102]. The inferior articular facets are modeled 1.5 mm thick and meshed with one layer of six-node wedge elements (Wed6). The nodes facing away from the contact side are connected to the adjacent rigid VHP vertebra. Parameters of the Neo-Hookean material for the facet cartilage follow literature values [24].

**Table 1:** Element types and material properties of the components of the final passive hybrid model.

Components	Material model and element type	Material properties	References
Vertebrae and endplates	Rigid bodies	$\varphi =1500\frac{kg}{m^{3}}$	[98, 112]
Superior articular process	Rigid bodies	$\varphi \approx 0\frac{kg}{m^{3}}$	
Inferior articular facets	Neo-Hookean (Wed6)	*E* = 35 *MPa*, *υ* = 0.4	[24]^a^
Nucleus pulposus	Yeoh (Hex8)	$\varphi =1000\frac{kg}{m^{3}}$,	[98]
		*c*_10_ = 0.20 *MPa*,	
		*c*_20_ = 0.20 *MPa*,	
		*c*_30_ = 6 *MPa*,	
		*k* = 0.18 *GPa* (*υ* = 0.499)	
Anulus ground substance	Mooney-Rivlin (Hex8)	$\varphi =1200\frac{kg}{m^{3}}$,	[26, 98]
		*c*_10_ = 0.18 *MPa*,	
		*c*_01_ = 0.045 *MPa*,	
		*k* = 2.25 *MPa* (*υ* = 0.41)	
Collagen fibers	Multi-point springs (tension only)	Custom nonlinear stress-strain curves (Fig 4B)	[26, 105]^a^
Ligaments	UWLigMat^b^	Combined for all springs describing a ligament	[105, 108, 113, 114]^a^
ALL	9 multi-point springs (tension only)	*ε*_*r*_ = −0.03, *ε*_*t*_ = 0.08, *k* = 4362 *N*	
PLL	5 multi-point springs (tension only)	*ε*_*r*_ = 0.12, *ε*_*t*_ = 0.23, *k* = 1451 *N*	
CL	10 axial springs^c^ (tension only)	*ε*_*r*_ = −0.55, *ε*_*t*_ = 0.95, *k* = 399 *N*	
LF	11 axial springs (tension only)	*ε*_*r*_ = −0.03, *ε*_*t*_ = 0.09, *k* = 330 *N*	
ISL	8 axial springs (tension only)	*ε*_*r*_ = −0.02, *ε*_*t*_ = 0.05, *k* = 20 *N*	
SSL	1 axial spring (tension only)	*ε*_*r*_ = −0.08, *ε*_*t*_ = 0.20, *k* = 28 *N*	
ITL	2 axial springs^c^ (tension only)	*ε*_*r*_ = −0.02, *ε*_*t*_ = 0.10, *k* = 298 *N*	

^a^ Properties based on references but have been modified.

^b^ Modified UWLigamentMaterial formulation (see Eq (3)).

^c^ On each side.

#### Springs

**Fig 3:**
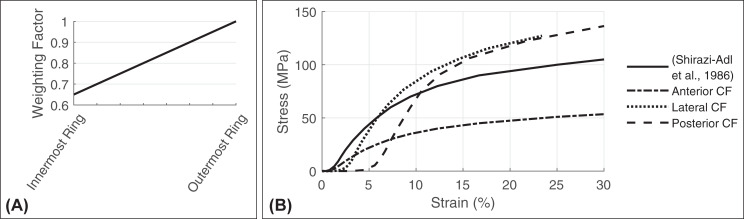
(A) Linear stiffness gradient and (B) stress strain curves of the collagen fibers. (B) The initial course of the stress-strain curve is taken from the literature [105] and adjusted during calibration process. A distinction is made between the three anatomical directions of the disc in which the CF are located: anterior, posterior, and lateral (compare Fig 2B).

For each disc, the CF are arranged in five rings in a crisscross pattern so that they are at angles of about ±32° to the end plates [23, 103, 104]. Using multi-point springs, the CF are connected to the external finite element nodes of the AGS lamellae. All CF run continuously from the respective inferior to the superior vertebra and are rigidly connected to these. In good agreement with the physiological structure [94], CF inclination angles increase towards the disc center due to the decrease of CF ring diameters. The fiber diameters are determined by the volume fraction of CF per lamella, the radial position, and the total number of CF rings. The total CF volume in the AF is assumed to be 16% [105, 106]. As the outer CF behave stiffer than the inner [28], their diameters have been weighted radially (Fig 3A). The nonlinear behavior of the tension only CF is described by the stress-strain relationship depicted by Shirazi-Adl et al. [105]. After calibration we apply three modified stress-strain curves to the CF using a custom material implementation in ArtiSynth (Fig 3B). CF are therefore automatically selected according to their positions in the AF (Fig 1C).

Seven intervertebral ligaments [107] are considered as springs: anterior longitudinal ligament (ALL), posterior longitudinal ligament (PLL), capsular ligaments (CL), ligamenta flava (LF), interspinous ligaments (ISL), supraspinous ligament (SSL) and intertransverse ligaments (ITL). Like CF, ALL and PLL are modeled as multi-point springs and are linked to the adjacent AGS nodes and thus are directly coupled with the deformation of the intervertebral discs [107]. All other ligaments are represented by axial springs, connecting two rigid bones. Spring locations, lines of action and quantities are based on anatomical origins and courses of the ligaments. Thus ligaments are represented by multiple springs [107–109]. To represent the tension only nonlinear ligament behavior and enable a pretensioning, by means of a reference stress *ε*_*r*_, we customized the implemented ligament model (UWLigamentMaterial) depicted in the University of Wisconsin knee model [110]. In the model’s initial state, the geometric spring length *l*_*G*_ describes the connection path from the beginning of the spring to its end point and *l*_0_ = *l*_*G*_(1+*ε*_*r*_) is an auxiliary length to achieve a pretensioning for *ε*_*r*_<0. While *l*_*G*_ and *l*_0_ are already determined during the model setup (*t* = 0), the actual spring length *l*(*t*) is calculated at each time step *t* to get the spring force *F*(*ε*(*t*)) from the case differentiation in formula (3).

$$F\left(\varepsilon (t)=\frac{l(t)-l_{0}}{l_{0}}\right)=\left\{ \begin{array}{cc} 0 & \varepsilon (t)\leq 0,\\ 0.125\;k\;{\varepsilon (t)}^{2}\varepsilon_{t}^{-1} & 0<\varepsilon (t)<\varepsilon_{t},\\ k(\varepsilon (t)-0.5\;\varepsilon_{t}) & \varepsilon (t)\geq \varepsilon_{t} \end{array}\right.$$ (3)

The transition strain from the low stiffness foot region to the stress-strain region with linear stiffness *k* is referred to as *ε*_*t*_. Initial parameters for *ε*_*r*_, *ε*_*t*_ and *k* have been chosen according to published material curves [105] and are adapted within the systematic calibration of FSU L4/5 (Table 1). Only the linear stiffnesses *k* have been scaled relative to FSU L4/5. To better match total and intervertebral range of motions with in vitro measurements, we adjusted some of the initial factors by Pintar et al. [111] during calibration. The adjusted factors are indicated with an ^a^ in Table 2.

**Table 2:** Stiffness scaling factors for LSS ligaments [111] with respect to L4/5 level.

Ligament	L1/2	L2/3	L3/4	L5/S1
ALL	0.85^a^	1.20^a^	1.05^a^	0.50^a^
PLL	0.66	1.42	0.41	0.84
CL	1.39	1.11	1.06	0.62^a^
LF	0.85	0.92	1.27	0.74
ISL	1.15	1.10	1.40^a^	1.03^a^
SSL	1.28	1.38	1.30^a^	0.71^a^
ITL	1.00	1.00	1.00	1.00

^a^ Factors altered during validation process.

### Boundary and loading conditions

In all multisegmental investigations the sacrum of the LSS model is fixed in space. By applying a six-dimensional spatial force (Wrench) to vertebra L1, force components set to zero and torques in a range of 0 to 12.5 Nm are exerted in all three anatomical planes (flexion/extension, lateral bending, and axial rotation). Since ligamentous LSS tends to buckle [14] we apply axial forces up to 2000 N with a compressive follower load (FL). Our FL is implemented as two sagittally symmetric tension only multi-point springs, each with a via-point attached to vertebrae L1 to S1 [57]. To simulate loads for the erect stand [67] and ensure comparability of results [63], we optimized the FL path by means of a search process (*patternsearch*() from the optimization toolbox, Matlab R2019b) with global settings within 547 steps. Each optimization step covers the sequence: (1) reset the simulation, (2) reposition via-points, (3) ramp and holding the FL at 1000 N, (4) retrieving four target values from ArtiSynth when a static state has been achieved. The target values are the absolute angular displacements around the Y-axis (Ry) stated by *θ*_*y*,*L*1_ and *θ*_*y*,*Ll*_ for the total range of motion (ROM) of L1 and the summed intervertebral ROM from L1 to L5, respectively. The magnitude of the summarized lumbosacral contact force vectors of left and right facet joints is *F*. All target values are weighted and combined in the cost function

$$f=w_{1}\theta_{y,Li}(p)+w_{2}\theta_{y,Ll}(p)+w_{3}F(p)$$ (4)

$$-1\leq p_{j}\leq 1\;\mathrm{for}\;j=1,2,\ldots ,K\;(K=6\;\mathrm{lumbosacral}\;\mathrm{levels})$$

that have been minimized where *p*_*j*_ represents the query point to return a via-point position value from the *j*-th interpolant. Thus, during optimization, the independent via-point positions on the 3D vertebral body surfaces are automatically varied in the posterior (*p*_*j*_<0)—anterior (*p*_*j*_>0) direction within a range of approximately ±5 mm from the sideward geometrical vertebral body centers. The effect of different weight factors *w*_*i*_ was tested and iteratively determined to $w_{1}=1\frac{1}{{}^{\circ}},\;w_{2}=0.5\frac{1}{{}^{\circ}}$, and $w_{3}=0.1\frac{1}{N}$, which best mimics an upright posture with almost unloaded facet joints. For a 1175 N FL the amounts of all intervertebral ROM Ry are below 0.49° (-0.12±0.31°) and *θ*_*y*,*L*1_ = -0.38°. With 0.74 N *F* is maximal at L4/5 level.

For comparison with literature values [115] relating to the lumbar spine (L1-L5) only, the influence of FSU L5/S1 is eliminated by locking vertebra L5. To simulate different maximum voluntary body postures [116] and validate intervertebral ROM and contact forces in facet joints (FF), we apply combined loading modes from Dreischarf et al. [97] (Table 3) to our model. When calibrating and validating FSU L4/5 solely, L5 is stationary, the FL is reduced to both vertebrae and the Wrench is applied to vertebra L4. All exerted loads are ramped to the maximum values to ensure stable and quasi-static simulations.

**Table 3:** Combined loading modes used for lumbar spine validation.

Posture	Compressive force via FL (N)	Moment (Nm)
Flexion	1175	7.5
Extension	500	7.5
Right lateral bending	700	7.8
Left axial rotation	720	5.5

### Verification

Besides the anticipated influences due to various load cases, material parameters, and geometries [117], other modeling decisions may also unintentionally affect the simulation results [118, 119]. In the context of hybrid modeling, our focus in verification is first on the finite element mesh and the material models of the L4/5 disc. Simplified models are used to minimize uncontrolled variables.

#### Mesh convergence study

For a mesh convergence study, a simplified model consisting of rigid vertebral bodies L4 and L5 with intermediate NP and AGS as one lamella is built. Based on this, in order to compare the influences of shape function (linear, quadratic), element type (Tet4, Tet10, Hex8, Hex20), and element density (Hex8: 364 nodes, 234 elements up to 8770 nodes, and 7560 elements), two, four, and six meshing variants are generated, respectively. All 12 variants are based on findings of additional upstream meshing studies not mentioned here. Material models and parameters for NP and AGS are initially taken from the literature [26, 98]. A sufficient mesh refinement is assumed if the calculated displacements and stresses change by 5% or less [118] with further refinement.

#### Pre-calibration

Aims of pre-calibration are the reduction of the modeling and calculation effort and a tuning of the initial mechanical response of the disc before validation. Based on our findings from the mesh convergence study, we extend the simplified FSU L4/5 by dividing the AGS into three to seven lamellae in radial direction and adding CF to specifically consider the following aspects in different configurations: (1) The hyperelastic material models for finite element NP and AGS and (2) the number of CF rings (Fig 2B). The reason for studying the material models in more detail is to set the characteristic behavior of the disc under axial compression and torsion. By using at least one of the common hyperelastic material models (Neo-Hookean, Mooney-Rivlin, and Yeoh) for the intervertebral disc, we target its characteristic nonlinear stiffening behavior under large axial compression with simultaneous high compliance under small bending moments. Due to the lack of other anatomical structures in the model, we only perform a qualitative comparison of the ROM and stiffness with in vitro literature data. To determine the least number of AGS lamellae, we consider the disc bulge and the computation time following Goel et al. [58] in a convergence study (changes < 3%). Because influences of CF, NP, and AGS can hardly be treated individually, the whole pre-calibration has been performed iteratively, but is reported only for the last passage.

### Calibration

The calibration of single FSU L4/5 follows the systematic in vitro reduction procedure of anatomical structures conducted by Heuer et al. [85] in reverse order. Accordingly, ten steps in which components are successively added are distinguished: (1) Only AGS between vertebral bodies L4 and L5 (2) AF, expansion of AGS by CF (3) intact disc with AF and NP (4) ALL (5) PLL (6) facet joints (7) CL (8) LF (9) ISL (10) SSL and ITL. At each step, the initial or already pre-calibrated material parameters are adjusted such that the ROM in flexion, extension, lateral bending, and axial rotation matches the published median values [85] best possible. While the variation of material parameters is executed using Matlab, both geometric adjustments (e.g., for the facet joints) and the final decision are done manually at each stage.

### Validation

Studies have shown that the kinematic system response, such as ROM, may not be sufficient for model validation [59, 97]. Thus, to choose model and simulation parameters that provide the best match to published experimental (Table 4) and numerical models data [47, 97, 120, 121], we use further mechanical responses from the single intact FSU L4/5: intradiscal pressure (IDP), FF, stiffness, ICR (instantaneous center of rotation), and center of rotation (COR). The COR are calculated as the mean of the two-dimensional centrode, the path traced by the ICR, to approximate the entire movement to a single point.

**Table 4:** Compilation of experimental studies included in calibration and validation.

Study	Objects of examination			Measurements					
	Lumbar levels	Sample size (male/female)	Mean age (range) in years	ROM		IDP	DB	FF	CR	K
Renner et al., 2007 [15]	L1-S1^a^	10 (3/7)	in vitro, 58 (41–73)	x/x					x/x
Wilke et al., 2001 [17]	L4/5	1 (1/0)	in vivo, 45		x/x				
Pearcy and Bogduk, 1988 [122]	L1-S1	10 (10/0)	in vivo,—(25–36)					x/x	
Heuer et al., 2007b [85]	L4/5	8 (4/4)	in vitro, 52 (38–59)	x/x					
Gardner-Morse and Stokes, 2004 [93]	L2/3, L4/5	8 (0/8)	in vitro, 37 (17–58)						x/x
Rohlmann et al., 2001 [115]	L1-L5	10 (8/2)	in vitro, 46 (18–74)	x/x	x/				
Andersson and Schultz, 1979 [123]	L1/2 –L4/5	16 (12/4)	in vitro, 55.5 (18–18)		x/x				
Berkson et al., 1979 [124]		42 (27/15)	in vitro, 42.8 (21–60)	x/	x/x				
Brinckmann and Grootenboer, 1991 [125]	T12/L1 –L4/5	15 (9/6)	in vitro, 31.3 (20–40)		x/x	x/			
Cuchanski et al., 2010 [126]	L1/2, L3/4, L5/S1	15 (2/13)	in vitro, 59 (34–70)			x/x			x/x
Guan et al., 2007 [127]	T12-S1^a^	10	in vitro, 50.6 (27–68)	x/x					
Haberl et al., 2004 [128]	L3/4, L4/5	10	in vitro, 55 (28–69)	x/				x/x	
Heuer et al., 2007a [129]	L4/5	8 (4/4)	in vitro, 52 (38–59)	x/x	x/x				
Heuer et al., 2008 [130]	L2/3	6 (2/4)	in vitro, 51 (38–59)	x/x		x/x			
Hirsch, 1955 [131]	L2/3, L4/5	15	in vitro,—(18–46)						x/x
Kotani et al., 2006 [132]	L1-S1 (L3/4, L4/L5)	7	in vitro, 71	x/x				x/x	
Nachemson et al., 1979 [133]	L1/2 –L5/S1	42 (27/15)	in vitro, 42.8 (21–60)	x/x	x/x				
Panjabi et al., 1994 [134]	L1-S1 (L2-S1)^a^	9 (9/0)	in vitro, 51 (35–62)	x/x					
Quint et al., 1998 [135]	L2-S2 (L4/5)	6	in vitro, 47.6±9.8	x/x					
Reuber et al, 1982 [136]	L1/2 –L4/5	14 (8/5)	in vitro, 57 (42–67			x/x			
Schultz et al., 1979 [137]	L1/2 –L4/5	42 (26/16)	in vitro, 43 (21–60)	x/	x/x				
Stokes and Gardner-Morse, 2016 [138]	L2/3, L4/5	8 (0/8)	in vitro, 37 (17–58)	x/x	x/x				
Wilson et al., 2006 [139]	L2-L5	4	in vitro, 76				x/x		
Yamamoto et al., 1989 [140]	L1-S1^a^	10	in vitro,—(25–63)	x/x					

x/x–Examined (measured) in study / used for current model calibration and validation

^a^ Examination of the entire LSS.

(DB: disc bulge, CR: instantaneous axis of rotation or center of rotation, K: stiffness of disc or FSU).

The LSS model is based on the findings of the validated FSU L4/5. Material parameters for discs and facet joints are transferred to the other levels without any modifications. Using adapted ligament stiffnesses (Table 2), the passive hybrid LSS is validated according to intervertebral and total ROM, IDP, FF, ICR, stiffness, and disc bulges by experimental in vitro studies (Table 4). Since only few complete in vitro data sets for a validation of the LSS exist, we compare our lumbar spine (L1-L5) with experimental data of Rohlmann et al. [115] and published numerical results [97].

## Results

### Verification of the FSU L4/5 model

#### Mesh convergence study

Meshing the L4/5 disc with different elements, number of elements, and shape functions results in diverging displacements, von Mises stresses, and calculation times. To calculate the IDP as a hydrostatic stress from the node normal stresses, hexahedral elements with a quadratic shape function (Hex20) proved to be inappropriate. Hex20 elements tend to buckle in case of soft incompressibility (using a restoring pressure based on a volume-based energy potential), while a hard incompressibility preventing this applies explicit boundary conditions which on one hand lead to instabilities and on the other hand set the volume changes of the elements to zero. Tetrahedral elements (Tet4 and Tet10) show an increased resistance to displacement in case of large deformations due to volumetric locking [112]. With a similar number of nodes, discs meshed with Hex8 (1869 nodes) or Tet4 (1816 nodes) show a similar computation time of approximately 11 s for one second of simulation. In context of the hybrid FSU, it turns out that for now only Hex8 elements are suitable to model the hyperelastic disc. Above a mesh density of 70 Hex8 elements per cubic centimeter, the stress and displacement solutions converge asymptotically, so that the maximum deviation is 3%. Coarser structured Hex8 meshes tend to underestimate the stiffness and tension of the simplified disc. Calculation time and element volume fraction correlate approximately positively linear.

#### Pre-calibration

**Fig 4:**
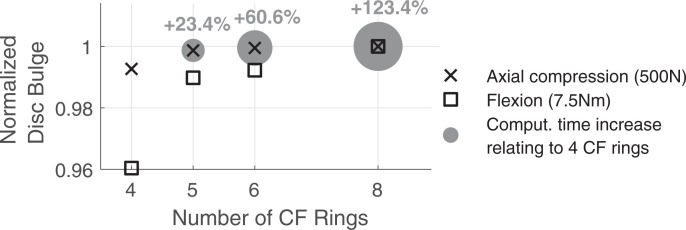
Sensitivity study of normalized disc bulge and computation times with four L4/5 disc models. The disc models vary in their number of CF rings and the associated AGS meshes (Fig 2B). Bulge is measured as the relative displacement of the points of measurement under load compared to the unloaded state. For axial compression disc bulge is averaged from anterior, posterior, and lateral positions. For flexion disc bulge is measured anteriorly only. The computation of one simulation second with four CF rings under increasing load took about 19 seconds on our reference system.

**Fig 5:**
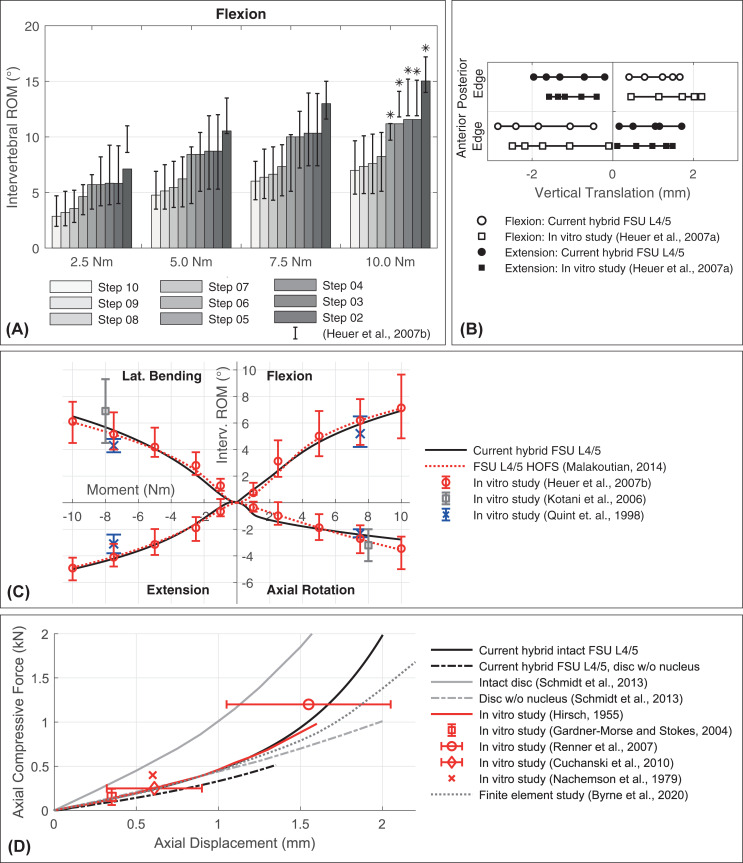
Comparison of kinematic responses for calibration (A) and validation (B-D) of intact FSU L4/5. (A) Intervertebral ROM in flexion when cumulatively adding anatomical components until FSU L4/5 is intact (step 10). Predicted values at four different loading magnitudes are compared to in vitro measured minimum and maximum values [85], represented by black error bars. *In vitro data from only three specimens due to multiple fails at 10.0 Nm. (B) Vertical component of the translational movement (Z-axis) of vertebra L4. The predicted translations are measured at the respective inferior posterior and anterior vertebral body edges and are compared to in vitro measured data [129]. In each case, five connected points visualize the pure moment amplitudes 1, 2.5, 5.0, 7.5, and 10 Nm starting from the origin. (C) The predicted nonlinear load-displacement curves of FSU L4/5 under pure moments are compared with results from three experimental in vitro studies using intact FSU [85, 132, 135]. The ROM of a HOFS implemented in ArtiSynth [47] are also shown. The HOFS replaces all structures connecting our rigid vertebrae L4 and L5. (D) Nonlinear displacement responses versus applied axial compression force. The stiffening behavior of our intact FSU L4/5 and a FSU L4/5 without NP is compared to in vitro data [15, 93, 126, 131, 133] and a numerical model from literature [120].

The mechanical responses of NP and AGS correlate directly with the material parameters of the hyperelastic constitutive models. For axial compressions exceeding 500 N, no nearly incompressible Mooney-Rivlin material parameter (*c*_01_, *c*_10_, *k*) combination for NP and AGS is found sufficiently simulating stiffening of the intervertebral disc [93] without significantly affecting flexibility in bending. Under large deformations, the simplifications of the Neo-Hookean and the Mooney-Rivlin material models has become discernible, which can be attributed to the linear stress strain relation with a constant slope [95, 99, 106]. By using eight CF rings and combining the Yeoh material model for NP and the Mooney-Rivlin material for AGS, we can simulate both the axial stiffening (Fig 5D) of the disc and its high flexibility under bending (Fig 5A and S1 Fig). With a higher number of CF rings, the mesh of the AGS is finer (see Fig 2B). For five or more CF rings in the L4/5 disc model, the predicted disc bulges and stiffnesses converge, so that the deviation between the results is less than 2% (Fig 4). However, compared to the disc with four CF rings, the computation time for five rings increases by 23.4%.

### Calibration and validation of the FSU L4/5 model

#### Kinematic responses

The intervertebral ROM in flexion for the stepwise calibration of the FSU L4/5 are shown in Fig 5A (for lateral bending, extension, and axial rotation in S1 Fig in the appendix) in comparison to in vitro measurements of Heuer et al. [85]. Motion patterns during the stepwise addition of FSU L4/5 components, such as NP and ligaments, correlate well with in vitro data. A decrease of ROM in flexion by adding facet joints (step 6) could not be observed. Decreases by adding the NP (step 3) and the CL (step 7) are largest with up to 23% at 10 Nm. With the restriction for 10 Nm from stage 6 and for 2.5 Nm from stage 3, all predicted ROM are within the experimentally measured variations.

The courses of rotation of vertebra L4 compared to in vitro data of intact FSU L4/5 are shown in Fig 5C. Except for small axial rotations (below 1.0 Nm with 1.071°) the simulated ROM are within measured in vitro ranges [85]. Moments for deflections of 1.0° around the principal axes are 1.40 Nm for lateral bending, 1.15 Nm for flexion, 1.88 Nm for extension, and 1.20 Nm for axial rotation. L4 rotates by 1.5° (Rx) under 1.95 Nm lateral bending. Compared to four in vitro tested FSU L4/5 for small rotations (<1.5°) without pre-load [138], our hybrid FSU behaves up to twice as flexible. Referring to the measurements of Heuer et al. [85], our model tends to overestimate small and underestimate large axial rotations. However, our lateral bending moment for 1° is in good agreement with the in vitro measurements. ROM at 2.5 to 10.0 Nm agree well with median values measured in vitro [85]. ROM results achieved with the ‘HeuerOffsetFrameSpring’ (HOFS) implemented in ArtiSynth [36, 47] correspond very well with the results of our intact hybrid FSU L4/5 (Fig 5C).

When loaded with pure moments, vertebrae can have translational motion components in addition to rotating ones. In Fig 5B the vertical translations of vertebra L4 are compared to experimental in vitro displacements in flexion and extension [129]. The biggest discrepancy (0.61 mm) in vertical direction of our hybrid model is posterior at a flexion of 10 Nm (1.64 mm). The horizontal translations also follow the in vitro values posteriorly with a maximum deviation of 0.6 mm under a 10 Nm flexion moment. Only anteriorly, in case of flexion moments greater than 2.5 Nm, the horizontal translation of L4 remains behind by up to 0.85 mm.

With increasing axial compression, intact FSU stiffen so that force-deformation curves increase exponentially (Fig 5D). Compared to in vitro measurements, the hybrid FSU L4/5 shows a similar response. Up to 0.9 mm axial displacement (0.39 kN) the curve runs approximately linear, which is fully consistent with in vitro measurements [93, 126, 131]. That the nonlinear axial stiffening is directly related to the presence of NP [141] can also be shown with our model.

**Fig 6:**
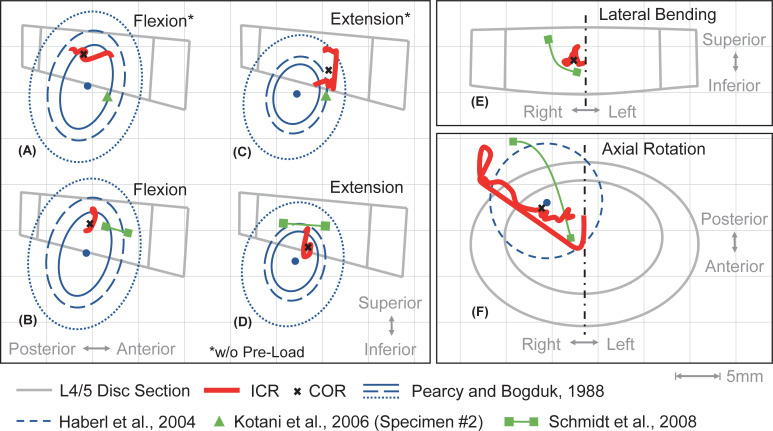
Centrodes traced by the ICR of vertebra L4 in flexion, extension, axial rotation, and lateral bending. As intersections of the instantaneous axes of rotation with the sagittal plane (A-D), the frontal plane at the center of L4/5 disc (E), or the superior endplate of vertebra L5 (F) the series of different ICR are shown as centrodes of motion (solid red line). To visualize the predicted centrodes with respect to an upright posture, the schematic L4/5 disc is shown in different sectional views: flexion and extension in lateral view (A-D), lateral bending (right) in dorsal view (E), and axial rotation (left) in caudal view (F). All applied moments are ramped from 0 to 10 Nm. For (B), (D), (E), and (F) L4 is additionally pre-loaded with 400 N by means of a FL. Mean ICR along the entire movements are visualized as COR (black cross). For comparison predicted ICR paths reported by Schmidt et al. [121], in vitro measured preoperative COR from Kotani et al. [132], the range of in vitro measured ICR with pre-load from Haberl et al. [128], and various confidence limits from in vivo ICR measurements from Pearcy and Bogduk [122] are transferred and visualized.

In addition to the ROM, the ICR of vertebrae are a fundamental part of the kinematic response and are shown for the three distinct planes in Fig 6. The centrodes traced by the ICR differ with and without pre-load. For extension (Fig 6C and 6D) the COR shifts posteriorly by 2,4 mm. With 400 N pre-load the ICR in flexion and extension (Fig 6B and 6D) are located within the 96% confidence limits and the COR are within twice the standard deviation of in vivo means measured by Pearcy and Bogduk [122]. However, our predicted ICR of L4 in reference to L5 tend to be in the superior portion of the in vivo confidence limits. With respect to increasing bending moments up to 10 Nm plus pre-load, L4 ICR are comparable with numerical results by Schmidt et al. [121]. In axial rotation, the ICR are located almost at the center of the L4/5 disc until the right facet joint gets in contact for moments above 0.8 Nm (Fig 6F). In agreement with Schmidt et al. [121] the ICR migrate towards the compressed facet joint and are located outside the disc. However, starting at about 3.7 Nm, the ICR travel back to the center of L4/5 disc, where mean ICR were measured in vitro [128]. With increasing moment, the superior side of the initially longitudinally oriented instantaneous axes of rotation tilt continuously to the posterior left. This and the locations of ICR agree well with the measurements of Haberl et al. [128].

#### Structural mechanics responses

**Fig 7:**
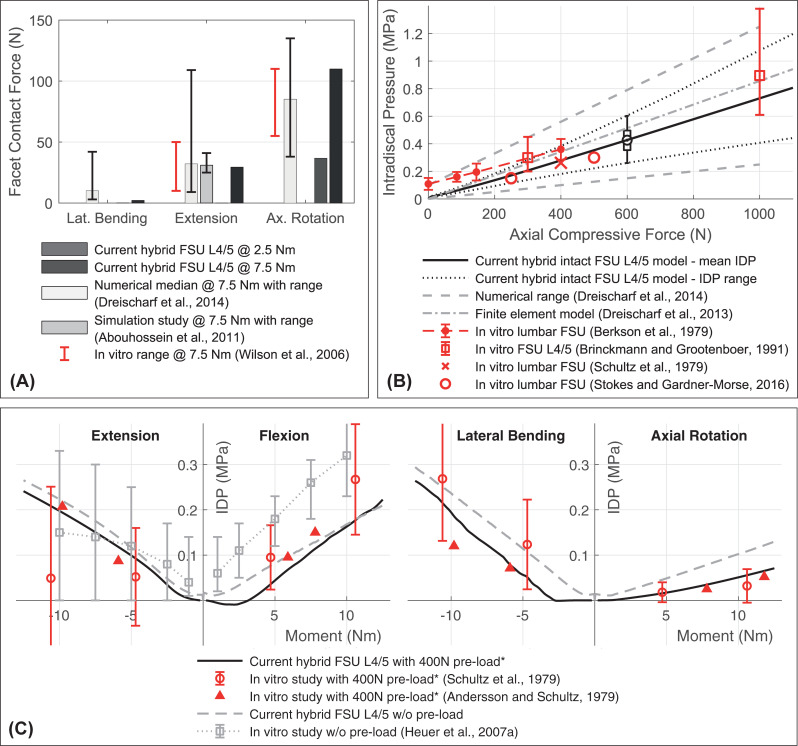
Comparison of structural mechanics responses for validation of FSU L4/5 (A-C). (A) Our hybrid FSU L4/5 is loaded with pure moments of 2.5 and 7.5 Nm. The predicted facet contact forces (3rd and 4th bar) are compared to in vitro measurements (red ranges) [139] and numerical values from literature [39, 97]. The reported numerical ranges represent minimal and maximal forces of all lumbar levels in eight models (1st bar) and ligament stiffness variations (2nd bar). (B) The predicted intradiscal pressure of our FSU L4/5 versus an axial compression force is compared to experimental in vitro data [124, 125, 137, 138] and values from published numerical models [97, 142]. The numerical range refers to the predictions by eight validated finite element models [97]. Since our IDP is calculated by averaging all nodal pressures in the NP [71], the resulting mean IDP (solid black line) and the scatter of individual pressure values (dotted lines) are shown. An exemplary pressure distribution is visualized as a box plot at 600 N. (C) The predicted IDP of our intact FSU L4/5 at eight loading conditions are compared to experimental in vitro data [123, 129, 137]. *The increasing IDP superimposed with 400 N pre-load are shown minus the initial mean compression-induced pressures. Subtracted initial IDP are 0.273 MPa for the current hybrid FSU L4/5 (solid black line) and 0.262 and 0.340 MPa, respectively, for the in vitro data [137] and [123].

Due to some prestressed ligaments, the mean IDP in unloaded condition is 0.013 MPa. With increasing axial compression via FL the computed IDP rises almost linearly (Fig 7B), which is in accordance with in vitro measurements [125]. Also, values and course correlate well with IDP measured in vitro at 250, 400, and 500 N [137,138]. By considering all nodes of the NP when calculating the mean IDP, we underestimated the IDP compared to other measurements [124,125]. All nodal pressures of our L4/5 NP are within the numerical range of published numerical models [97]. Agreement with an in vivo measurement during relaxed standing by Wilke et al. [17] (0.43–0.50 MPa) is plausible (610 to 697 N). The cross-sectional L4/5 disc area of the current model is 16.7 cm^2^. Following the calculation described by Dreischarf et al. [142] for estimating the compressive force from the IDP, the correction factor for our model is 0.85 at 500 N. Fig 7C illustrates the mean IDP for moments alone and combined with 400 N compression. For better comparison, the increasing IDP under pre-load are given from their initial compressed state. The predicted initial pressure is 0.273 MPa. The variance of in vitro data in some cases is high [129, 137]. However, except for flexion without pre-load the predicted IDP agree well with published in vitro data.

In case of flexion, the facet joint surfaces of the FSU L4/5 do not touch. Fig 7A shows the FF for the three other postures under 2.5 and 7.5 Nm, because directly measured in vitro FF are limited [139]. Our comparison proves that the total FF are in the range of other published numerical models and in vitro measurements. For lateral bending and extension at 2.5 Nm FF are 0 N. FF greater than zero are measured for lateral bending, extension, and axial rotation at 3.0, 3.6, and 0.9 Nm, respectively.

### Validation of the lumbosacral spine model

#### Kinematic responses

**Fig 8:**
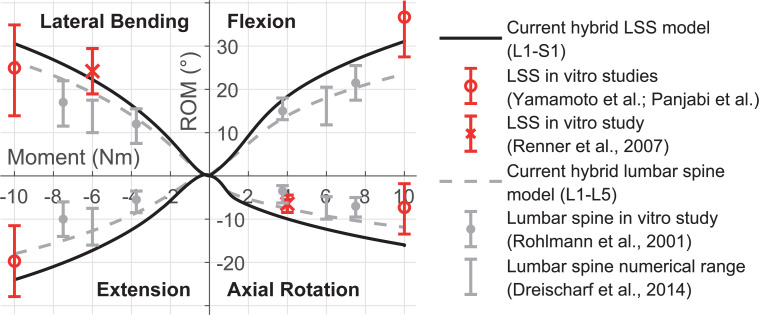
Nonlinear load-displacement curves of LSS (L1-S1) and lumbar spine (L1-L5) under pure moments. The predicted total ROM are compared to in vitro measurements [15, 115, 140] and the numerical range of eight published simulation models [97].

For pure moments of 10 Nm around the principal axes, the total ROM of vertebra L1 in relation to the fixed sacrum are 30.9° for lateral bending, 31.39° for flexion, 24.28° for extension, and 16.1° for axial rotation. The total ROM from L1 to L5 (lumbar spine) at 10 Nm are 26.65° for lateral bending, 23.76° for flexion, 18.18° for extension, and 11.87° for axial rotation. Compared to in vitro measurements, the total ROM curves are depicted in Fig 8. Except in case of axial rotation, the total ROM of our LSS model is within the standard deviations of in vitro studies [15, 134, 140]. The axial rotation exceeds the standard deviation by 16%. With fixed L5, the total ROM of the lumbar spine matches with the range of published numerical results [97]. Merely for lateral bending moments above 4 Nm our ROM are higher. Compared to a lumbar spine in vitro study [115] our model tends to a larger ROM except for flexion. For 7.5 Nm lateral bending the lumbar spine total ROM is at the upper end of the standard deviation measured in vitro.

**Fig 9:**
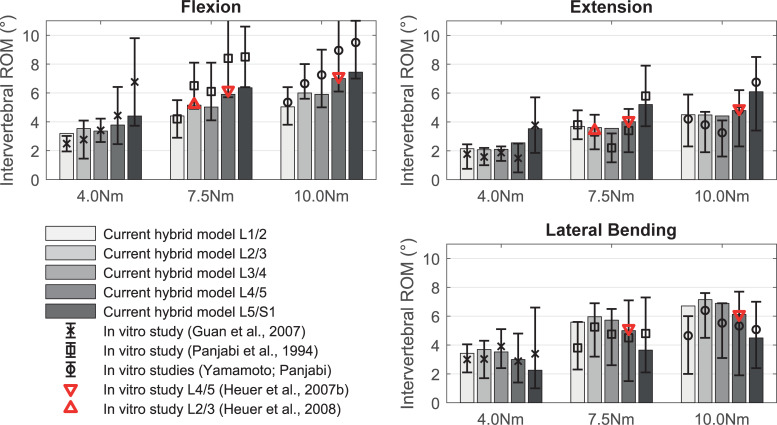
Intervertebral range of motion of the lumbosacral spine model loaded with pure moments. Comparison of our predicted intervertebral ROM in flexion, extension, and lateral bending (for axial rotation see S2 Fig in the appendix) with experimental in vitro data for completely tested LSS [127, 134, 140] and solely tested FSU [85, 130]. For comparison at 10 Nm the data of Yamamoto et al. and Panjabi et al. are combined.

Compared to in vitro measurements, the intervertebral ROM of the LSS are visualized in Fig 9. The in vitro experiments of Panjabi et al. [134] and Yamamoto et al. [140] were performed under additional pre-loads of up to 150 N for technical reasons. Our model does not show significant differences in intervertebral ROM between 0 and 150 N pre-load. The mean intervertebral ROM of the LSS model with standard deviations ($\bar{x}\pm SD$) for lateral bending, flexion, extension, and axial rotation at 4 Nm are 3.18±0.58°, 3.65±0.47°, 2.48±0.62°, and 1.77±0.16°, respectively. Intervertebral ROM for 7.5 Nm are 5.18±0.93°, 5.37±0.77°, 4.01±0.69°, and 2.40±0.23° as well as for 10 Nm 6.27±1.06°, 6.28±0.95°, 4.86±0.70°, and 2.78±0.27°. Thus, the intervertebral ROM for levels L1/2 to L3/4 are within the standard deviations of the in vitro measurements. The axial intervertebral ROM of level L4/5 and L5/S1 are above the in vitro standard deviations, but deviate for L4/5 by a maximum of 8% from the median values of intact FSU L4/5 [85] (Fig 5C). Our LSS model shows qualitatively similar trends described in the literature regarding different intervertebral ROM (Fig 9). Only in case of extension deviations are noted for level L3/4 and in case of lateral bending for L5/S1. In flexion, the intervertebral ROM are at the lower end of the standard deviations measured for the whole LSS. Increased mobility in flexion and extension of FSU L4/5 compared to the superior levels has also been observed in vivo [116]. The increased variance of the L5/S1 level measured by Pearcy [116] become apparent as well.

**Fig 10:**
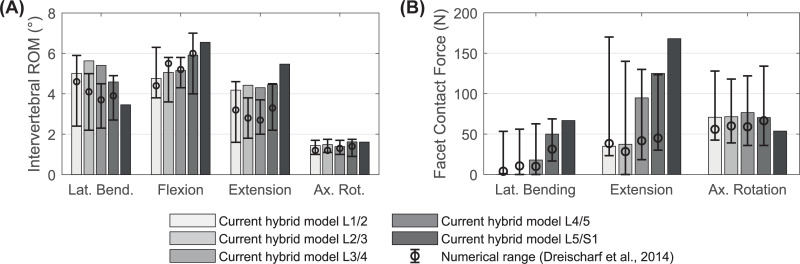
Mechanical responses of the hybrid LSS under combined loading modes (Table 3). (A) Predicted intervertebral ROM and (B) facet contact forces are compared to the median values and value ranges (error bars) of six published numerical lumbar spine models [97].

Compared to our lumbar spine model without pre-load, the total ROM decrease for a moment of 7.5 Nm plus 280 N pre-load by 0.5% to 22.4° in lateral bending, and by 6.7% to 9.80° in axial rotation. These findings correlate well with the in vitro behavior observed by Rohlmann et al. [115], that the influences of a pre-load of 280 N for lateral bending, flexion and extension are minor. For axial rotation, the in vitro ROM decrease is on average four times higher. Our model responds differently for flexion and extension: a pre-load increases the ROM by 6% and 8% respectively by unloading the ligaments. A higher FL (1.2 kN) stiffens the lumbar spine and the ROM in flexion is reduced by 1.5%. Compared to published simulation models, the intervertebral ROM for flexion and axial rotation show good agreement for all lumbar spine levels (Fig 10A). However, FSU L2/3 and L3/4 overestimate ROM for lateral bending and extension.

**Fig 11:**
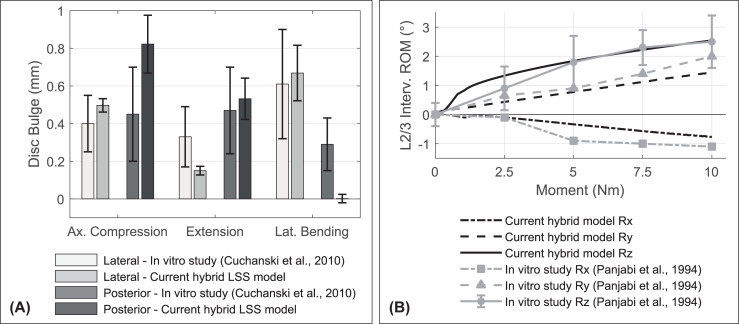
(A) Mean bulges of intervertebral discs and (B) load-displacement curves under pure axial rotation moment. (A) Comparison of L1/2 to L5/S1 disc bulges (mean ± standard deviation) with in vitro measurements of all lumbosacral levels [126]. Disc bulges are measured posteriorly and laterally. (B) Comparison of predicted coupled motion of hybrid FSU L2/3 with in vitro measurements [134].

Besides the nonlinear load displacement curves (Figs 5C and 8), the hybrid model shows typical motion coupling effects of intact FSU described in the literature [134, 137]. For an axial applied moment, Fig 11B illustrates additional rotations around the X- and Y-axis (Rx and Ry) to the main movement around the Z-axis (Rz). Directions of movement are most pronounced in axial rotation and amplitudes are very similar to those reported in vitro. For lateral bending, the coupled motion of our model matches in vitro intervertebral ROM as well [134]. No significant couplings are observed in flexion and extension [135].

Under an axial compressive FL, the force-displacement curves of the entire LSS and the FSU L4/5 (Fig 5D) are almost identical in their shape. Up to 350 N (2.31 mm) the LSS curve rises linearly and axial displacements of 4.13 mm and 5.21 mm are reached at 1 kN and 2 kN. Axial stiffnesses of the five FSU vary. The FSU L4/5 has the lowest axial compression resistance (646 N/mm at 1 kN). The total LSS stiffnesses are 89 N/mm for 250 N, 114.2 N/mm for 500 N, 152.3 N/mm for 1 kN, and 165 N/mm for 1.2 kN. For 1.2 kN compression, the individual stiffnesses are: 718 N/mm for L4/5, 790 N/mm for L2/3, 798 N/mm for L3/4, 950 N/mm for L5/S1, and 1063 N/mm for L1/2. Axial displacements measured in vitro with a 1.2 kN FL [15] are in very good agreement with our results. The largest axial displacement was also found in vitro at levels L2/3, L3/4, and L4/5 with an average of 1.5±0.55 mm (800±293 N/mm). In the same study the levels L1/2 and L5/S1 showed the highest stiffnesses with an average of 960±370 N/mm (1.25±0.4 mm). Another in vitro study measured individually axial displacements for all lumbosacral planes under 250 N compression but showed great variance (0.61±0.29 mm) [126]. With 0.52±0.093 mm for all FSU of our LSS we are within the standard deviations of the in vitro study.

A comparison of the ICR and COR of our hybrid LSS with in vivo measurements [122] shows a high agreement in their locations for flexion and extension. The COR for flexion at 400 N pre-load tends to be in the anterior, inferior range of the in vivo confidence limits (see Fig 6).

#### Structural mechanics responses

As in case of the sole validation of FSU L4/5 (see Fig 7B), the IDP profiles are linear with increasing axial compression for all LSS levels. At 1 kN compression the IDP decrease from L1/2 level to L4/5: 0.867 MPa, 0.842 MPa, 0.820 MPa, and 0.737 MPa. The mean pressure in NP at L5/S1 level is 0.762 MPa. Brinckmann and Grootenboer [125] in vitro measured IDP at 1 kN for FSU L2/3 and L4/5 (1.08±0.20 and 0.88±0.26 MPa) and observed an IDP reduction inferiorly. For 400 N compression predicted IDP from FSU L1/2 to L5/S1 are 0.33, 0.32, 0.32, 0.29, and 0.30 MPa. The magnitude and inferior decrease are in good agreement with other in vitro measurements [133].

The predicted contact forces in the facet joints of the LSS vary for lateral bending and extension between the different levels. Compared to elsewhere numerically calculated FF of the lumbar spine [97], Fig 10B shows the FF of our LSS for the same load cases. Facet joint surfaces do not touch during flexion. Regarding lateral bending and axial rotation, our model corresponds well to the published mean values. With 125.1 N in extension, the FF of FSU L4/5 is at the upper end of the numerical range of values.

Local disc bulge amplitude differences under 500 N axial compression or 7.5 Nm bending moment correspond well with in vitro measured values of six FSU L2/3 [130]. According to these load cases, the maximum calculated bulges on level L2/3 are at the following locations: For flexion anterior (1.61 mm), extension posterior (0.98 mm), lateral bending right lateral (1.93 mm), axial rotation anterior (0.09 mm), and for axial compression posterior (1.03 mm). Compared to an older in vitro study [136] we overestimate posterior disc bulge between 400–800 N. Lateral disc bulge (400 N: 0.70±0.06 mm, 800 N: 1.05±0.12 mm) are in the upper range of in vitro standard deviations. Amplitudes and locations of inward bulging show good agreement with in vitro measurements as well [130]: posterior for flexion (-0.51 mm), anterior for extension anterior (-0.61 mm), and right lateral for lateral bending (-1.91 mm) and axial rotation (-0.37 mm). Under axial compression our intervertebral discs do not bulge inwards. For all LSS levels most calculated disc bulges are within the standard deviations of an in vitro study [126] (Fig 11A). Merely the posterior disc bulges at 2.5 Nm lateral bending are significantly below in vitro results.

## Discussion

Hybrid model simulations are a viable but still less established alternative to MB and implicit FE models used separately or coupled, which can have often discussed drawbacks in more detailed investigations of the physiological interplay of skeletal muscles and structural mechanics. The aim of this study was to develop, validate, and report an open access hybrid FE-MB simulation model that represents an average, non-specific ligamentous LSS (lumbosacral spine) of a middle age man. To also keep the underlying anatomy data comprehensible, our LSS is based on the widely referenced VHP (Visible Humane Project). The segmentation, modeling, and meshing performed is not limited to the commercial programs we used in upstream processing steps. Since the final model design in ArtiSynth is done via open file formats such as OBJ or text files, any upstream tools can be used to generate them. The basic challenge was to model a LSS in a way that the mechanical responses are comparable to a FE model simulation, while keeping the hybrid modeling simple and robust enough to serve as a basis for an active simulation model with integrated trunk muscles. To comprehensively test the biomechanical validity of the model, we used a variety of experimental in vivo and in vitro studies, combining and discussing their data. Overall, our current approach shows that the coupling of a FE and MB simulation model will not remain the only way to investigate flexible bodies such as intervertebral discs structurally under in vivo like loading modes in the future.

In terms of patient-specific statements, the current model is limited [143]. By generalizing [87–90, 92] the basic anatomy [83] and combining various published material data (Table 1), our model represents a physiological LSS without individual characteristics. This must be taken into account when using the original image data in the future. Nevertheless, the freely available dataset of the Male VHP provides comprehensive information regarding the mass-inertia characteristics of the whole body [144] as well as, for example, the cross-sectional areas and courses of the muscles. By adapting the morphology and lumbar lordosis our LSS shows high similarities to other numerical models [19, 57]. However, even small geometrical variations may have a significant influence on mechanical responses [25]. Possible specific correlations arising between the modeled geometry and the material parameters used have not been explicitly examined in this study.

Consistent with the literature data used, our examinations are conducted quasi-statically by slowly increasing all loads from an unloaded state. Linear and nonlinear damping behaviors, as well as acceleration effects could thus be excluded, but play an important role in dynamic studies [39]. Since we currently use an implicit solver to solve the discrete-time sequences, dynamic solutions are unconditionally stable. However, to achieve convergence even at higher or abrupt accelerations keeping the material nonlinearities and contacts, the time steps currently limited to 0.01 s, the damping terms, and the solver may be adapted. To also increase efficiency in the future, an explicit solver may be considered. Apart from stability and efficiency, the validity of the model parameters described here cannot be conclusively assessed in a dynamic context. Since time-dependent poro- and viscoelastic effects are neglected as well, creep or swelling behavior of the intervertebral discs cannot be investigated [26, 145].

Due to missing complete experimental in vitro data sets for all mechanical responses of the LSS [36, 37], a consistent comparison could not be performed. To calibrate and validate our LSS simulation model in terms of reported mechanical responses, we combine or average various studies, regardless of their differing samples, experimental setups, and procedures. By restricting the analysis to the load cases described, we have not included all the data from the references given in Table 4. It should be noted that such procedure does not prove model validity, but only supports their probability.

The stepwise calibration of the anatomical FSU L4/5 components, based on in vitro measurements of Heuer et al. [85, 129], allows to adjust their individual effects on the overall mechanical response. Intervertebral ROM (range of motion) and IDP (intradiscal pressure) show high accordance at all levels with intact discs. Without and with pre-load small in vitro intervertebral ROM of the LSS are characterized partly by large differences between studies [85, 134, 138, 140]. However, especially for higher loads (>3 Nm, >300 N), more relevant in investigations, our model shows good alignment with in vitro measurements for concentrated and combined loads. There are wide variations in the ligament force-elongation curves used in the literature [108]. Compared to the fundamental descriptions of Shirazi-Adl et al. [105] our linear stiffnesses are significantly lower for ISL, SSL, LF, and ITL, similar for PLL and higher for ALL. Like CF (collagen fibers), ALL, and PLL are directly coupled to the bulging of the intervertebral disc by means of multi-point springs. The pretensioning of the ligaments determined in the stepwise calibration of the FSU L4/5 intervertebral ROM show good agreement with published values [8, 85]. The ligament material descriptions for the other lumbosacral levels are scattered. Therefore, only the linear stiffness values of FSU L4/5 have been scaled using the in vitro measured data of Pintar et al. [111] and partially adjusted (Table 2) to fit in vitro ROM. In vitro tests revealed that iliolumbar ligaments, which we do not model at this point, reduce the intervertebral ROM of FSU L5/S1 in flexion and extension by up to 5° [140].

The LSS simulation model includes 12047 nodes for the five discs and 562 nodes for the inferior articular facets. The sum of all springs for collagen fibers and ligaments is 2292. Despite the significant geometric simplification of the FE discs, they account for the largest share of the computing times. For the entire LSS model, under combined loads and contacting facet joints, including visualization at each time step, simulations run with about 0.40 time steps per seconds on our reference system. On systems with higher performance, this value increases considerably. As we show in our convergence study (Fig 4), simplifications of the disc increase the performance, which should be intensively investigated in following studies and evaluated against the needed degree of accuracy. In this study, accuracy was our primary concern. Regarding a comparison of the calculation time of lumbar spine FE models, we could not find any published values. Due to the computational efficiency of a plain MB model, its necessity, especially in the context of extensive optimizations, is considered indispensable. An advantage of the hybrid modeling shown is the capability to represent the relevant mechanical properties of the LSS easily and comparatively accurately without using complex and possibly limiting intervertebral joints [46, 50, 146]. The use of ArtiSynth provides the capability to match hybrid FSU and ‘HeuerOffsetFrameSprings’ (Fig 5C) in their mechanical response and, depending on the application, to substitute them in order to optimize computational efficiency or to refine structural details.

The simplifying assumption that CF runs approximately ±32° in the anulus fibrosus as well as the assignment of three force-elongation curves can have an influence on the calculated fiber strain, IDP, and FSU kinematics [147]. Based on the descriptions of Bruehlmann et al. [148] and by modeling the CF by means of continuous multi-point springs, our CF slide frictionless through the anulus ground substance via nodes. However, the exact physiological behavior of CF in the ground substance matrix is still a matter of debate and may be better characterized by its alignment and elongation under load [130].

Many simulation models considerably simplify or neglect nonlinear effects of intact FSU [146]. By using fiber-reinforced intervertebral discs modeled by almost incompressible Mooney-Rivlin and Yeoh materials, physiological nonlinear responses of the spinal column are properly predicted for different loading conditions. In contrast to models using two-term Mooney-Rivlin materials for the entire disc [26, 98], we could only simulate physiological stiffening at high axial compressions (>300 N) by means of a hyperelastic material model based on a strain-energy formulation with 3^rd^ order polynomial (Yeoh) for the nucleus pulposus.

Assuming that tissues of the LSS behave much more flexible than bones [22, 149] and that the calculation times can be reduced [76], all vertebral bones and cartilaginous end plates are modeled as rigid bodies. Furthermore, we assume that the mechanical relevance of cartilage is reduced as it becomes thinner with aging, calcifies [150] and is replaced by bone [96]. This can lead to an overestimation of the stresses at the transition from rigid bone to the connecting deformable discs and ligaments [39, 143]. At the almost right-angled edges of the intervertebral discs, we identify local stress concentrations. Compared to disc bulges, the shift of the end plates into the respective vertebrae is described as small [136]. However, this inward bulges cannot be represented by the hybrid LSS, which may lead to an overestimation of the IDP [94]. For flexion we identify an underestimation of the IDP in our model. This is caused by calculating the IDP by means of all nodes of the posteriorly shifted nucleus pulposus and thus not directly considering the anteriorly compressed anulus ground substance. A corresponding evaluation only in the anterior section of the nucleus pulposus results in an IDP that is on average 30% higher. Moreover, by the way of modeling we neglect that the behavior of a non-degenerated disc’s center resembles an enclosed fluid [151].

Muscles, ligaments, and the facet joints may influence the instantaneous centers of rotation of vertebrae [122], whose movement patterns have been identified as an indicator for low back pain [152] and pathological conditions such as lumbosacral instabilities [41, 153] or spondylolisthesis [51]. Due to inaccurate geometric data, we idealized the facet joints as smooth and curved surfaces. Because real facet joint surfaces have complex curvatures and no ideal contact, their physiological behavior is only approximated [39]. Facet joints can be a common source of spinal diseases [59], inter alia the facet joint poses have been specifically defined for each FSU according to the VHP anatomy data and in vitro measurements [90, 92]. The interconnection of facets and vertebral bodies is rigid. For instance, in the case of large loads structural effects cannot be considered. The results for the maximum contact forces and motion couplings, however, especially in axial rotation in which the vertebrae move helically due to the facet joint contacts, correspond well with those described in the literature [154]. Also, the path of the FL (follower load) can have a non-negligible influence on FF (contact forces in the facet joints). Since in vivo load distributions can only be estimated indirectly and depend on numerous boundary conditions, assumptions for the compressive force components transmitted through the facet joints in upright posture range from 0 to 19% (in physiological state) [107]. Without considering FF as a target variable in our FL path optimization procedure, the facet joints bear 0 to 3% of the axial compressive load (3% at level L5/S1 and 0% at level L2/3). Therefore, unlike conventional optimization strategies [63], we minimize intervertebral rotations along with FF to approximate the influence of the FL on the load sharing between vertebral levels during calibration and validation.

Inevitably, it is standard practice in computing-intensive implicit FE simulations to assume simplifying boundary conditions and loading modes (FL plus pure moments), which can, however, severely affect the estimation of internal loads. As is common in MB models [36] our hybrid LSS could also be integrated into a musculoskeletal whole-body model to consider mass and inertial effects of the upper body. To better estimate probable in vivo lumbar loads and to fully take advantage of the hybrid model simulation approach using ArtiSynth, an extension with tensile muscles is our next step. Therefore, muscle activities in the context of muscle redundancy problems [155] must be determined in an inverse manner [156] to load and stabilize the LSS. To evaluate vertebral stresses and strains that may alter mechanical responses and be associated with pain, the replacement of certain rigid vertebrae with meshed vertebrae can be aimed at in the future as well. Overall, the further development of the hybrid LSS simulation model has the potential to provide a new, self-contained tool that enables the estimation of spinal responses under various loads to improve the biomechanical understanding for treating causes of pain. Also, the described modeling procedure itself can serve as a tool by means of which, for example, patient-specific or pathological models may be built.

## Supporting information

S1 Fig Intervertebral range of motion of FSU L4/5 at four different loading magnitudes. Predicted ROM in lateral bending, extension, and axial rotation from anatomically reduced to intact FSU L4/5 are compared to minimum and maximum values measured in vitro [85], represented by error bars. *In vitro measurements from only three specimens due to multiple fails at 10.0 Nm.
(TIF)

S2 Fig Intervertebral ROM of the lumbosacral spine model loaded with pure moments. Comparison of predicted intervertebral ROM in axial rotation with experimental in vitro data for completely tested LSS [134, 140] and sole FSU [85]. For comparison at 10 Nm the data of Yamamoto et al. and Panjabi et al. are combined.
(TIF)
