# Supplementary figures and images for: Calibration and validation of a novel hybrid model of the lumbosacral spine in ArtiSynth–The passive structures
Source: PLoS One. 2021 Apr 26;16(4):e0250456. doi: 10.1371/journal.pone.0250456 (PMC8075237; doi:10.1371/journal.pone.0250456)

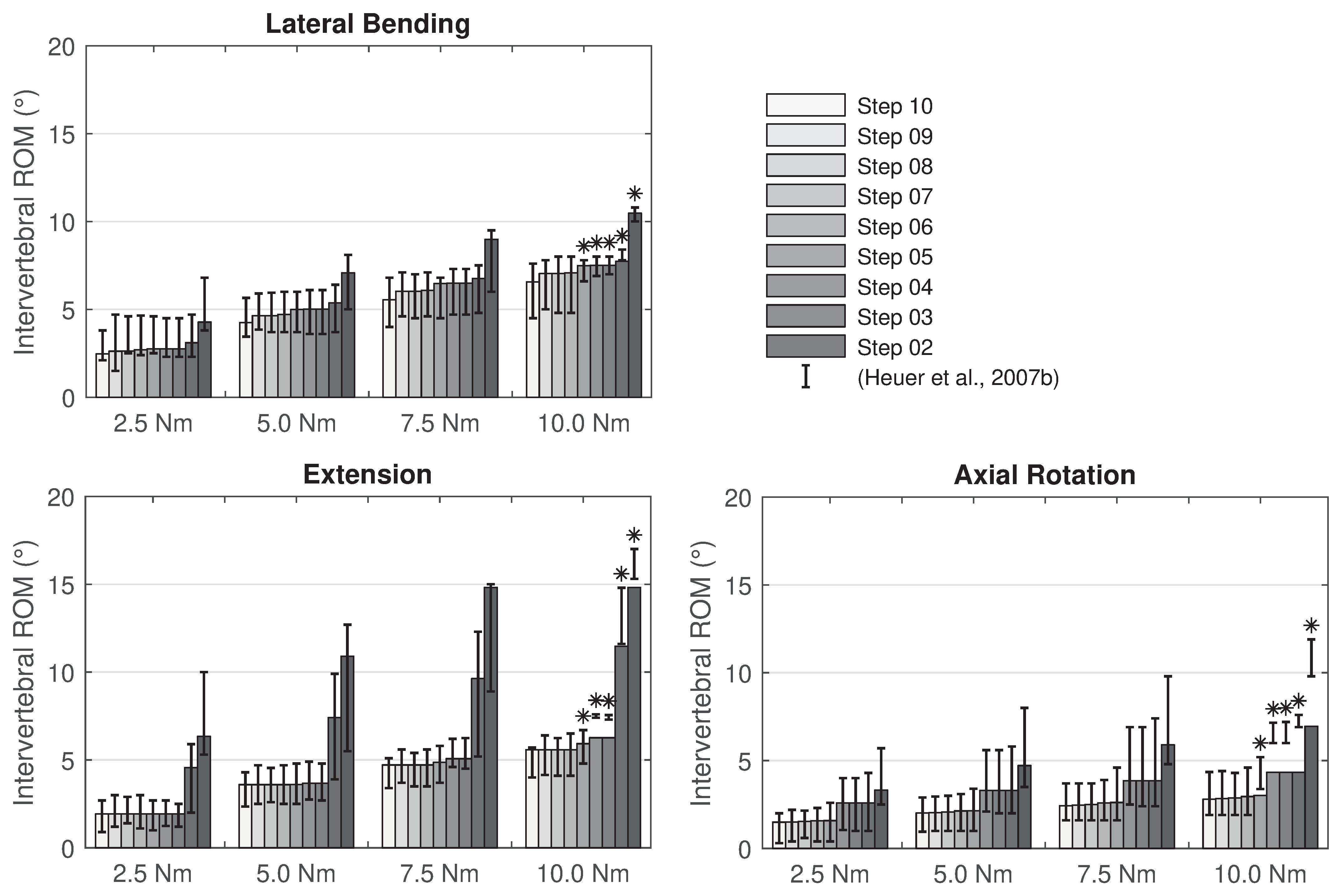

Supplement: S1 Fig — Predicted ROM in lateral bending, extension, and axial rotation from anatomically reduced to intact FSU L4/5 are compared to minimum and maximum values measured in vitro [85], represented by error bars. *In vitro measurements from only three specimens due to multiple fails at 10.0 Nm. (TIF) [file pone.0250456.s001.tif]

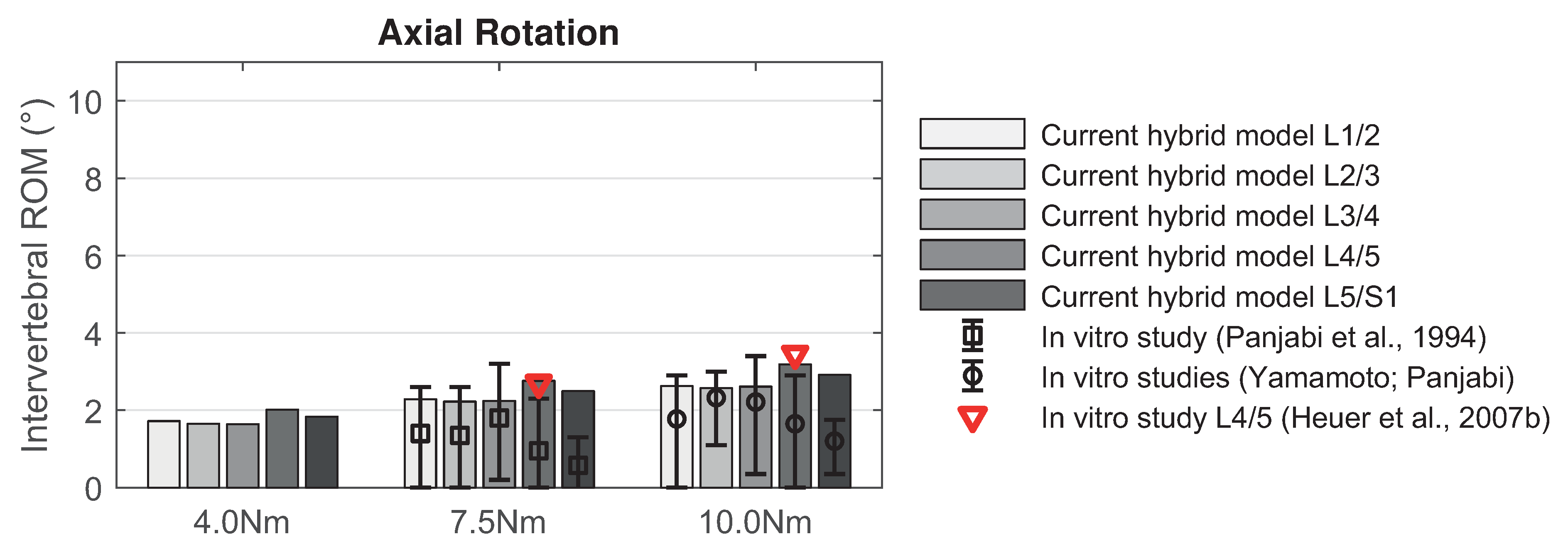

Supplement: S2 Fig — Comparison of predicted intervertebral ROM in axial rotation with experimental in vitro data for completely tested LSS [134, 140] and sole FSU [85]. For comparison at 10 Nm the data of Yamamoto et al. and Panjabi et al. are combined. (TIF) [file pone.0250456.s002.tif]
